# Children’s Emotions Upon the Shedding of the First Primary Tooth From Parents’ Perspective

**DOI:** 10.1155/ijod/5942652

**Published:** 2026-01-29

**Authors:** Lamis D. Rajab, Lyn A. E. El-Smadi

**Affiliations:** ^1^ Department of Pediatric and Preventive Dentistry, School of Dentistry, The University of Jordan, Amman, Jordan, ju.edu.jo

**Keywords:** community pediatric dentistry, emotions, Jordan, primary teeth, Tooth Fairy

## Abstract

**Background:**

The loss of the first primary tooth is a significant early biological milestone in a child’s development. As a novel experience, it often triggers a complex mix of excitement, confusion, and apprehension. Parents should focus on providing support to ensure this transition is viewed as a positive and reassuring event.

**Aims:**

To report children’s different emotions upon losing their first primary tooth and possible factors that may influence these emotions.

**Study Design:**

An online cross‐sectional survey utilized social media and recruited 988 parents of children aged 5‐8 years. Eligibility required that the child had experienced the exfoliation of at least one primary tooth.

**Methods:**

Besides parental demographics, the study used a questionnaire to evaluate child‐specific variables: sex, duration of tooth wiggle, and emotional state during exfoliation. History of traumatic dental injuries or caries‐related visits and witnesses of siblings’ tooth exfoliation were also recorded.

**Statistics:**

Descriptive statistics, chi‐square tests, and logistic regression tests were used.

**Results:**

Of all parents, 54.6% reported positive emotions, and 30.4% reported negative emotions. Significantly higher positive emotions were found in children getting dental check‐up visits (OR = 1.49, 95% CI: 1.07–2.07), and when the child witnessed tooth exfoliation in older siblings (odd ratio (OR) = 1.73, 95% confidence interval (CI): 1.29–2.32). Significantly more negative emotions were found in children attending public schools (OR = 1.41, 95% CI: 1.02–1.95), speaking one language (OR = 1.92, 95% CI: 1.29–2.87), having two or more siblings (OR = 1.42, 95% CI: 1.02–2.98), and having a negative attitude to a dental visit (OR = 1.68, 95% CI: 1.23–2.30).

**Main Conclusions:**

The majority of children associate the loss of their first primary tooth with positive emotions. Conversely, approximately one‐third experience negative emotions. The emotional response is significantly influenced by child‐related factors and previous dental visits.

## 1. Introduction

Primary teeth represent a substantial part of the body, and children are born without them [1]. Primary tooth development starts before birth and is fully completed by approximately the fourth postnatal year [2, 3]. Emergence of primary teeth begins around 4–8 months of age with the emergence of the lower incisors and is completed at around 24–36 months of age upon the appearance of the second primary molars [4]. By 7 years of age, the primary central and lateral incisors have been or are in the process of being replaced by their permanent successors, and the permanent first molars have already erupted. The remaining primary dentition usually includes the canine and first and second molars of both arches [5]. The loss of primary teeth might be considered one of the first biological changes children consciously experience [6].

Emotions are a group of physiological and cognitive processes triggered by different experiences and thoughts. There are many definitions of the term “emotion,” with no consensus on a specific definition. However, emotions are usually recognized to be composed of neural circuits, neurobiological responses, and a feeling that motivates and organizes cognition and action [7]. The emotions associated with losing the first primary tooth might be related to what it means to children; it can lead to positive emotions as children may perceive losing it as a sign of growing up [8]. However, according to Capps and Carlin [1], something inherently anxiety‐provoking is associated with the loss of a primary tooth, and it is related to the unclear status of these teeth in the human body. Considering the fact that primary teeth are not present at birth, and then they make a complete set of teeth followed by their loss one after another, this contributes to an ambiguous status of primary teeth to the child [1]. Patcas et al. [6] found that only one in five children experienced negative emotions at the loss of the first primary tooth and stated that these negative emotions seem less influenceable than positive emotions. Parental negative responses to negative emotions like distress and sadness expressed by the child were correlated with negative socioemotional outcomes for children [9–11]. Negative responses to children’s negative emotions include distress reactions and minimization reactions [11]. More positive responses are encouraged, such as expressive encouragement and emotion‐focused reactions. Understanding emotions associated with the natural loss of the first primary tooth and the influencing factors, together with an acknowledgment of family traditions regarding the shed tooth, can provide an opportunity for dentists to bond with the young child. Positive and empathetic interactions in this age group can help prevent dental anxiety and many negative consequences [12]. Although the recommendation for the first dental visit is to occur at age one to prevent early childhood caries [13], many parents, in a recent study, think this first visit should happen much later, even at age 6 [14]. Thus, dentists may not see pediatric patients until they are almost or have reached tooth‐shedding age, underscoring the sensitive nature of these early interactions [12]. Since dental anxiety initially develops in early childhood [15], and while a child’s first interactions with dental professionals are particularly influential, negative experiences during these crucial years may contribute to the development of dental anxiety that lasts an entire lifetime [12].

Traditions about baby teeth are found worldwide and in many historical eras [1]. In Middle Eastern countries, there is a tradition of throwing a baby tooth up into the sky, to the sun, or to Allah [16]. In some European countries, however, the Tooth Fairy might be used in some families after the shedding of the primary tooth. The Tooth Fairy is a folkloric character that has been considered a prominent member of the Fairy family and is committed in her professional career to exchanging exfoliated primary teeth for money [17]. The tooth disposal ritual was practiced as a rite of passage to ease the transition from early to later childhood, to counteract anxiety, trauma, or pain experienced by children of tooth‐shedding age, and to honor spiritual or religious beliefs that are thought to protect the child and aid their healthy development [12].

As the psychological impact of the shedding of primary teeth has not earned enough scholarly attention [6], and in an attempt to bring attention to the importance of knowledge of possible influences of emotions experienced by children during the shedding of the first primary tooth before its replacement by the permanent successor on the child’s emotional development, the objectives of this study were to: (1) investigate the association between the experience of typical tooth shedding and emotional response among children aged 5‐8 years in Jordan from their parent’s perspective, (2) the possible factors that may influence the reported emotions, and (3) assess the traditions related to the lost primary tooth and factors influencing the use of Tooth Fairy.

## 2. Materials and Methods

Ethical approval was obtained from the Department of Pediatric Dentistry at the School of Dentistry and from the Council of the School of Postgraduate Studies at the University of Jordan. Also, the study was reviewed and approved by the Institutional Review Board (IRB) of Jordan University Hospital. It was stated clearly that participation in the study is voluntary and that responses will be completely anonymous. Consent was obtained through a question proving the approval to participate in the study.

This cross‐sectional epidemiological survey targeted parents of children of both sexes aged between 5 and 8 years in Jordan who at least had one shed primary tooth. This age group was chosen to increase the likelihood of participating in the study. Parents from all parts of Jordan who could read and write the Arabic language and had internet access were invited to participate in the survey. This study was built on an online survey using a questionnaire. Over a 1000 messages with the electronic link of the questionnaire were sent to Jordanian parents through social media (Facebook, WhatsApp, and Messenger). Responses were collected over 2 weeks in June 2020. Parents were reached from the authors’ social groups that were found to be specifically created for parents (mothers or fathers) from different cities in Jordan. Parents contacted were asked to pass the electronic link of the questionnaire to other parents with the same inclusion criteria. A message about sending the survey to others appeared to participants after they had submitted their responses.

The sample size was estimated using G. power 3.03 [18]. Because of the lack of enough previous studies, a low effect size of (0.10) was considered to ensure that the sample is sufficient for a larger effect size (at the power of 0.80 at the 0.05 two‐tailed level of significance) using logistic regression; z‐tests, the minimum sample needed was 721. The sample approach was increased to consider possible refusal and incomplete responses.

This study used the same questionnaire developed and used by Patcas et al. [6], who, upon request, shared it with the investigators. The original questionnaire was in German and translated into English, then sent back to Patcas [6] to check for errors. Finally, it was translated into Arabic with slight modifications to be adapted for the Jordanian culture. The tool has been translated utilizing the WHO guideline for tool translation and adaptation [19]. Furthermore, face validity was conducted utilizing the expert panel of three experts in the field of dentistry who have the experience, knowledge, and expertise in research and the body of science.

The questionnaire contained 32 questions and was composed of several parts. The first part contained 17 questions about child‐related factors, including the child’s age, sex, school type attended by the child, city of residence, languages spoken by the child, number of siblings, number of siblings older than the child, parents with the child in the same household. Also, this part contained questions related to the dental factors regarding the child’s frequency of tooth brushing, number of dental visits, reasons for dental visits, child’s last dental visit, and child’s attitude to a dental appointment. Other questions related to the child’s experience of shedding the first primary tooth, including the age of the child when the first tooth was lost, the number of primary teeth lost, the storing of lost teeth, how the first tooth fell off, the time of noticeable tooth wiggle, if the child experienced tooth exfoliation in older siblings, and the emotions experienced by the child when the first tooth started to wiggle and when this tooth was lost. The second part of the questionnaire comprised three questions asked about the parent’s relation to the child (the parent who completed the survey) and the parents’ educational level. The third part has one question that asks parents about their child’s experience of losing a primary tooth and what they tell their child when the primary teeth are lost. Parents can choose from a list of categories only one answer that best describes their child’s emotions of joy, pride, being scared, fearful and anxious, ashamed, and sad, or no specific positive or negative emotion. An accurate measurement of expressed emotions is complex and requires advanced methods such as the facial action coding system [20]. Because it can be challenging to distinguish between different emotions, the method applied by Patcas [6] was adopted in that it was chosen to group the various emotions into categories of positive emotions (joy and pride) and negative emotions (being scared, fearful and anxious, ashamed, and sad) for statistical analysis. The responses of neither positive nor negative emotions were excluded from the analysis. The last part was about Tooth Fairy, 11 questions were about who tells the child about the Tooth Fairy, from where parents know about it, if the Tooth Fairy was used during the mother’s childhood or father’s childhood, what the Tooth Fairy puts in place of the tooth, what the Tooth Fairy does with the teeth she takes, what the amount of money the Tooth Fairy leaves for the child depends on, if the child believes in the Tooth Fairy, and if there are any other fictional characters the child believes in.

A pilot study was conducted on 30 participants before commencing the main study. The sample has been recruited using a convenience sampling technique. The participants used in the pilot study were recruited from the same setting used to recruit the sample included in the analysis. The inclusion criteria applied to the pilot sample were the same as the study sample. The purpose of the pilot was to obtain information related to clarity, understandability, cultural appropriateness, and time needed to fill out the survey. Minor changes were made upon parents’ comments, but that did not require further validity investigation. The time required to complete the survey was around 10 min.

## 3. Statistical Analysis

Data analysis was performed using the SPSS Statistical Software Package SPSS for Windows release 16.0 (SPSS Inc., Chicago, IL, USA). All analyses were conducted with two‐sided tests. Descriptive analyses were conducted. Statistical tests were run for the association between positive and negative emotions and other independent variables. The responses of neither positive nor negative emotions were excluded from the analysis. Bivariate associations were evaluated using the chi‐square Pearson correlations test. After variables were dichotomized, multivariate binary logistic regression models were computed to examine influencing factors and odds ratios (ORs) (with 95% confidence intervals (CIs). Two models calculated the odds of children’s positive and negative emotions being influenced by parent‐ and child‐related factors. The third model determined the odds of the prevalence of telling the child about the Tooth Fairy being influenced by parent‐ and child‐related factors. To check the validity and reliability of the translation of the questionnaire, the confirmatory factor analysis (CFA) was carried out. The level of significance for all tests was set at *p*  < 0.05.

## 4. Results

A total number of 988 questionnaires were completed online. Of these questionnaires, 88.8% (*N* = 877) were completed by children’s mothers, 6.8% (*N* = 67) by fathers, and only 2.1% (*N* = 21) were completed by both parents. The children’s mean age was 6.8.

Table 1 shows the demographic characteristics of the study sample and the child‐related factors. The majority of children had lost their first primary tooth at the age of 5 and above but less than 7 years. A quarter of parents reported that primary teeth are kept after exfoliation, mostly by children themselves. More than half of parents reported that their children had their first primary tooth exfoliated on its own without any help. Also, Table 1 shows different responses that were given from parents’ beliefs and practices regarding the child losing the first primary tooth. More than half of parents linked the loss of the primary tooth to the child’s growth; about one‐third made their children put the primary tooth under the pillow or let their children throw the exfoliated primary tooth to the sun and ask the sun to replace it with deer’s teeth. A minority of parents (15.3%) said that they tell their children about the Tooth Fairy.

**Table 1:** Demographics of the study sample.

Variable		*N* (%)	Variable		*N* (%)
Sex	Male	492 (49.8)	Child’s daily frequency of tooth‐brushing	Frequent	404 (40.9)
	Female	496 (50.2)		Infrequent	584 (59.1)
School type	Private	594 (60.1)	Number of dental visits	Zero	195 (19.)
	International	156 (15.8)		One	198 (20.)
	Public	238 (24.1)		Two	190 (19.2)
City of residency	Amman (the capital)	629 (63.7)		Three	137 (13.9)
	Other cities	359 (36.3)		Four	83 (8.4)
Number of languages spoken by the child	One language	766 (77.5)		More than four	185 (18.7)
	Two or more languages	222 (22.5)	Cause of previous dental visit (*N* = 793)	Accident	89 (11.2)
Siblings of the child	One or no siblings	300 (30.4)		Caries	536 (67.6)
	Two siblings	327 (33.1)		Extraction	52 (6.6)
	More than two siblings	361 (36.5)		Checkup	247 (31.1)
Siblings older than the child	One or no siblings	605 (61.2)	Child’s attitude to a dental treatment (*N* = 793)	Positive	176 (22.2)
	Two siblings	180 (18.2)		Negative	281 (35.4)
	More than two siblings	203 (20.5)		Neutral	336 (42.4)
Parent/s with the child in the same household	Mother and father	925 (93)	Age when first tooth was lost	Less than 5	77 (7.8)
	Mother or father	56 (5.7)		5 years and above but less than 6 years	345 (34.9)
	Other	7 (0.7)		6 years and above but less than 7 years	453 (45.9)
Mother’s education	Mid school or high school	114 (11.5)		Between 7 and 8 years	113 (11.4)
	College diploma or bachelor’s degree	726 (73.5)	The first tooth fell off	On its own	588 (59.5)
	Master’s degree and doctoral degree	148 (15.0)		With the parents/teacher help	265 (26.8)
Father’s education	Mid school or high school	230 (23.3)		By the child	66 (6.7)
	College diploma or bachelor’s degree	554 (56.1)		By a dentist	69 (7.0)
	Master’s degree or doctoral degree	204 (20.7)	Time of noticeable tooth wiggle	Few days	397 (40.2)
Traditions related to the loss of the first primary tooth	Throw it to the sun	294 (29.8)		More than 1 week	386 (39.1)
	Exchange tooth for money from under the pillow	303 (30.6)		More than 1 month	205 (20.7)
	Talking to the child about the importance of permanent teeth	385 (39)	Experienced tooth exfoliation in older siblings/friends	Yes	665 (67.3)
	The exfoliation of teeth means the child has grown	560 (56.7)		No	323 (32.7)
	Other (e.g., to bury it in the ground)	7 (0.7)	Tooth Fairy visit	Parents tell the child about the Tooth Fairy	151 (15.3)

Table 2 shows information related to the use of the Tooth Fairy; mostly, the mother tells the child about it. Most parents who use the Tooth Fairy with their children tell their children that the Tooth Fairy swaps the tooth from under the pillow with a gift or money. More than half of parents leave up to five Jordanian dinars (around 7 USD, 5 GBP, 6 EUR) for the child as a gift from the Tooth Fairy.

**Table 2:** Information on the use of Tooth Fairy (*N* = 151).

Characteristic		*N* (%)	Characteristic		*N* (%)
Who tells the child about a Tooth Fairy?	Mother	92 (60.9)	What does the Tooth Fairy do with teeth she takes?	Good teeth become stars in the sky	20 (13.2)
	Father	1 (0.7)		Decayed teeth to use them to build a castle	9 (6)
	Both parents	35 (23.2)		Decayed teeth cannot be used	9 (6)
	Other (aunt, sister, …etc.)	32 (2.5)		Do not specify what the Tooth Fairy will do with teeth she takes	97 (64.2)
From where parents know about Tooth Fairy	Parents	48 (31.8)		Other (e.g., use it as a necklace, build a house with it)	16 (10.6)
	TV	36 (23.8)	What does the amount of money the Tooth Fairy will leave for the child depends on?	Child’s behavior: good behavior means more money	20 (13.2)
	Social media	10 (6.6)		The lost tooth number (first tooth more money)	10 (6.6)
	Relatives/friends	50 (33)		Quality of the tooth (decayed teeth less money)	3 (2)
	Books	4 (2.6)		Does not depend on a specific thing	100 (66.2)
	School	4 (2.6)		Grades in school	3 (2)
Was the Tooth Fairy also used during the mother’s childhood?	Yes	62 (41.1)		Not applicable	15 (9.9)
	No	89 (58.9)	How much money does the Tooth Fairy leave?	The child does not get money for the lost tooth	10 (6.6)
Was the Tooth Fairy also used during the father’s childhood?	Yes	36 (23.8)		Less than 1 Jordanian dinar (JD)	13 (8.6)
	No	115 (76.5)		From 1 to 5 JDs	83 (55)
What does the Tooth Fairy put in place of the tooth?	Will take the tooth without replacing it with anything	7 (4.7)		From 6 to 10 JDs	15 (9.9)
				From 11 to 20 JDs	8 (5.3)
	Swaps the tooth for a gift or money	106 (70.2)		More than 20 JDs	2 (1.3)
				Did not specify	20 (13.2)
	Puts a gift or money next to the tooth	20 (13.2)	Does the child believe in the Tooth Fairy?	Yes	96 (63.6)
	Leaves a letter with money inside	10 (6.6)		No	21 (13.9)
	Did not specify	8 (5.3)	Does the child believe in the imaginary characters?	Yes	326 (33)
				No	662 (67)

Table 3 shows the distribution of reported emotions experienced by children upon the loss of the first primary tooth. Above one‐half of the parents reported that their children had positive emotions, frequently joy, while one‐third indicated that their children experienced negative emotions, mostly fear and anxiety. Only 15% of the parents reported that their children had neither positive nor negative emotions.

**Table 3:** Reported emotions experienced by children upon the loss of the first primary tooth.

Type of emotion	*N*	%	Reported emotion	*N*	%
Positive emotions	539	54.6	Joy	516	95.7
	—	—	Pride	141	26.2
Negative emotions	300	30.4	Being scared	69	23
	—	—	Fear or anxiety	229	76.3
	—	—	Being ashamed	35	11.7
	—	—	Sadness	48	16
Neither positive nor negative emotions	149	15.1		—	—

Table 4 shows the association between variables and emotions related to tooth‐shedding experiences and also to the use of the Tooth Fairy. School type, number of languages spoken by the child, number of siblings of the child, number of siblings older than the child, father’s level of education, child’s daily frequency of tooth brushing, number of dental visits, a child having a check‐up related dental visit, child’s attitude to dental treatment, age when the first tooth was lost, tooth exfoliation experienced in older siblings/friends all have a statistically significant association with positive emotions experienced (*p*  < 0.01). Also, school type, number of languages spoken by the child, number of siblings of the child, father’s level of education, a child having a check‐up related dental visit, child’s attitude to dental treatment, tooth exfoliation was not experienced in older siblings/friends had a significant association with negative emotions as well (*p*  < 0.01). There was a significant association between the prevalence of telling the child about the Tooth Fairy and type of school, city of residence, number of languages spoken by the child, number of siblings of the child and mother’s level of education, the frequency of tooth brushing, caries‐related dental visits, check‐up‐related dentist visits, and the child’s attitude toward dental treatment (*p*  < 0.01).

**Table 4:** Difference in demographics, dental factors and tooth shedding experience related to emotions and the use of Tooth Fairy (*N* = 988).

Variable		*N*	Positive emotions		*p*	Negative emotions		*p*	Tooth fairy prevalence		*p*
			*N*	%		*N*	%		*N*	%	
Sex	Male	492	260	52.8	0.3	148	30.1	0.8	80	16.3	0.4
	Female	496	279	56.3		152	30.6		71	14.3	

School type	Private	594	316	53.2	0.000	184	31	0.000	88	14.8	0.000
	International	156	118	75.6		21	13.5		51	32.7	
	Public	238	105	44.1		95	39.9		12	5	

City of residence	Amman	629	349	55.5	0.5	189	30	0.8	112	17.8	0.004
	Other cities	359	190	52.9	—	111	30.9	—	39	10.9	—

Number of languages spoken by the child	One language	766	381	49.7	0.000	261	34.1	0.000	86	11.2	0.000
	Two or more languages	222	158	71.2		39	17.6		65	29.3	

Siblings of the child	One or no siblings	300	190	63.3	0.000	73	24.3	0.004	72	24	0.000
	Two siblings	327	180	55		96	29.4		43	13.1	
	More than two siblings	361	169	46.8		131	36.3		36	10	

Siblings older than the child	One or no siblings	605	360	59.5	0.000	177	29.3	0.4	101	16.7	0.3
	Two siblings	180	86	47.8		54	30		28	15.6	
	More than two siblings	203	93	45.8		69	34		22	10.8	

Parents with the child in the same household	Mother and father	925	505	54.6	0.6	283	30.6	0.6	137	14.8	0.2
	Mother or father	56	29	51.8		16	28.6		12	23.1	
	Other	7	5	71.4		1	14.3		2	28.6	

Mother’s education	Mid school or high school	114	51	44.7	0.1	42	36.8	0.3	21	18.4	0.007
	College diploma or bachelor’s degree	726	406	55.9		215	29.6		96	13.2	—
	Master’s degree and doctoral degree	148	82	55.4		43	29.1		34	23	—

Father’s education	Mid school or high school	230	110	47.8	0.004	79	34.3	0.02	30	13	0.09
	College diploma or bachelor’s degree	554	299	54		175	31.6		80	14.4	—
	Master’s degree or doctoral degree	204	130	63.7		46	22.5		41	20.1	—

Child’s daily frequency of tooth brushing	Frequent	404	341	58.4	0.004	171	29.3	0.4	112	19.2	0.000
	Infrequent	584	198	49	—	129	31.9	—	39	9.7	—

Number of dental visits	Zero	195	92	47.2	0.03	62	31.8	0.2	23	11.8	0.5
	One	198	103	52	—	69	34.8	—	31	15.7	—
	Two	190	110	57.9	—	55	28.9	—	29	15.3	—
	Three	137	69	50.4	—	48	35	—	21	15.3	—
	Four	83	49	59	—	22	26.5	—	11	13.3	—
	More than four	185	116	62.7	—	44	23.8	—	36	19.5	—

Previous accident‐related dental visit (*N* = 793)	Yes	89	55	61.8	0.3	27	30.3	1.0	17	19.1	0.4
	No	704	392	55.7	—	211	30	—	111	15.7	—

Previous caries‐related dental visit (*N* = 793)	Yes	536	300	56	0.8	155	28.9	0.4	71	13.2	0.001
	No	257	147	57.2	—	83	32.3	—	57	22.2	—

Previous extraction‐related dental visit (*N* = 793)	Yes	52	25	48.1	0.2	21	40.4	0.1	9	17	0.9
	No	741	422	57	—	217	29.3	—	119	16	—

Previous check up‐related dental visit (*N* = 793)	Yes	247	166	67.2	0.000	57	23.1	0.004	55	22.3	0.002
	No	546	281	51.5	—	181	33.2	—	73	13.4	—

Child’s attitude to a dental treatment (*N* = 793)	Positive	176	128	72.7	0.000	31	17.6	0.000	39	22.2	0.008
	Negative	281	134	47.7	—	115	40.9	—	32	11.4	—
	Neutral	336	185	55.1	—	92	27.4	—	57	17	—

Age when first tooth was lost	Less than 5	77	33	42.9	0.006	28	36.4	0.1	18	23.4	0.2
	5 years and above but less than 6 years	345	179	51.9	—	114	33	—	55	15.9	—
	6 years and above but less than 7 years	453	272	60	—	120	26.5	—	63	13.9	—
	Between 7 and 8 years	113	55	48.7	—	38	33.6	—	15	13.3	—

The first tooth fell off	On its own	588	313	53.2	0.1	173	29.4	0.2	88	15	0.1
	With the parents/teacher help	265	149	56.2	—	84	31.7	—	35	13.2	—
	By the child	66	44	66.7	—	16	24.2	—	11	16.7	—
	By a dentist	69	33	47.8	—	27	39.1	—	17	24.6	—

Time of noticeable tooth wiggle	Few days	397	203	51.1	0.2	120	30.2	1.0	53	13.4	0.2
	More than 1 week	386	221	57.3	—	118	30.6	—	60	15.5	—
	More than 1 month	205	115	56.1	—	62	30.2	—	38	18.5	—

Experienced tooth exfoliation in older siblings/friends	Yes	665	384	57.7	0.004	182	27.4	0.003	94	14.1	0.2
	No	323	281	24.3	—	483	72.6	—	57	17.6	—

Table 5 shows the predictors of positive and negative emotions. The odds of experiencing positive emotions were higher by around two times in children who speak two or more languages, 1.40 times in children who had one or no siblings, 1.5 times in children who had a dental visit and the cause was for a routine check‐up, two times in children who had a positive attitude toward a dental appointment, 1.4 times in children who lost their teeth at the age of 6 years and above, and by 1.7 times in children who had witnessed primary tooth exfoliation in older siblings or friends. Likewise, children had higher odds of experiencing negative emotions by 1.4 times if they were attending public schools, by two times if speaking only one language, by 1.4 times if having more than two siblings, and by 1.7 times if having a negative attitude to a dental appointment.

**Table 5:** Logistic regression analysis predicting positive and negative emotions upon shedding of the first primary tooth (*N* = 988).

Independent variables		Positive emotions experienced by the child *N* (%)	Unadjusted odds ratio (95% CI)	*p*	Adjusted odds ratio (95% CI)	*p*	Negative emotions experienced by the child *N* (%)	Unadjusted odds ratio (95% CI)	*p*	Adjusted odds ratio (95% CI)	
School type	Private or international	434 (57.9%)	1.74 (1.30–2.33)	**0.000**	1.28 (0.92–1.76)	0.14	205 (27.3%)	1.77 (1.30–2.40)	**0.000**	1.41 (1.02–1.95)	**0.04**
	Public	105 (44.1%)					95 (39.9%)				

Number of languages spoken by the child	One language	381 (49.7%)	2.50 (1.81–3.45)	**0.000**	1.90 (1.34–2.70)	**0.000**	39 (17.6%)	2.43 (1.66–3.53)	**0.000**	1.92 (1.29–2.87)	**0.001**
	Two or more languages	158 (71.2%)					261 (34.1%)				

Siblings of the child	One or no siblings	190 (63.3%)	1.68 (1.27–2.22)	**0.000**	1.33 (0.94–1.88)	0.11	73 (24.3%)	1.53 (1.13–2.08)	**0.007**	1.42 (1.02–1.98)	**0.04**
	Two or more siblings	349 (50.7%)					227 (33%)				

Siblings older than the child	One or no siblings	360 (59.5%)	1.68 (1.29–2.17)	**0.000**	1.40 (1.02–1.91)	**0.035**	Not statistically significant				
	Two or more siblings	179 (46.7%)									

Father’s education	Mid school or high school	110 (47.8%)	1.42 (1.06–1.91)	**0.02**	1.03 (0.75–1.42)	0.86	79 (34.3%)	1.27 (0.93–1.74)	0.13	Not statistically significant	
	Higher degree	429 (56.6%)					221 (29.2%)				

Child’s daily frequency of tooth brushing	Frequent	341 (58.4%)	1.46 (1.13–1.88)	**0.004**	1.10 (0.83–1.45)	0.504	Not statistically significant				
	Infrequent	198 (49%)									

Number of dental visits	Zero or one dental visit	195 (49.6%)	1.39 (1.08–1.80)	**0.01**	1.13 (0.85–1.50)	0.41	Not statistically significant				
	Two or more dental visits	344 (57.8%)									

Previous check up‐related dental visit	Yes	166 (67.2%)	2.02 (1.50–2.74)	**0.000**	1.49 (1.07–2.07)	**0.019**	57 (23.1%)	1.63 (1.17–2.27)	**0.004**	1.26 (0.89–1.80)	0.20
	No	373 (50.3%)					243 (32.8%)				

Child has a positive attitude to a dental appointment	Yes	128 (72.7%)	2.60 (1.82–3.73)	**0.000**	2.02 (1.35–3.00)	**0.001**	31 (17.6%)	2.32 (1.53–3.51)	**0.000**	1.67 (1.07–2.61)	**0.02**
	No	411 (50.6%)					269 (33.1%)				

Child has a negative attitude to a dental appointment	Yes	134 (47.7%)	1.47 (1.12–1.94)	**0.006**	1.24 (0.91–1.69)	0.17	115 (40.9%)	1.96 (1.46–2.62)	**0.000**	1.68 (1.23–2.30)	**0.001**
	No	405 (57.3%)					185 (26.2%)				

Age when first tooth was lost	Less than 6 years	212 (50.2%)	1.36 (1.05–1.75)	**0.019**	1.39 (1.06–1.83)	**0.017**	Not statistically significant				
	6 years and above	327 (57.8%)									

Experienced tooth exfoliation in older siblings/friend	Yes	384 (57.7%)	1.48 (1.13–1.94)	**0.004**	1.73 (1.29–2.32)	**0.000**	182 (27.4%)	1.53 (1.15–2.03)	**0.003**	1.74 (1.29–2.35)	**0.000**
	No	155 (48%)					118 (36.5%)				

*Note:* Statistically significant results are shown in bold (*p* < 0.05).

Table 6 shows the factors predicting the prevalence of parents telling their children about the Tooth Fairy. Parents were more likely to tell their children about the Tooth Fairy with higher odds of 2.6 times when their children are attending private or international schools, by 1.5 times if children living in Amman, the capital of Jordan, by two times if children speak two or more languages, by 1.7 times when children have one or no siblings, and also by 1.6 times when children brush their teeth regularly.

**Table 6:** Determinants of the prevalence of telling the child about Tooth Fairy (*N* = 988).

Independent variables		Tell the child about Tooth Fairy		Unadjusted odds ratio (95% CI)	*p*	Adjusted odds ratio (95% CI)	*p*
		Yes *N* (%)	No *N* (%)				
School type	Private or international	139 (18.5%)	611 (81.5%)	4.29 (2.33–7.88)	**0.000**	2.63 (1.39–4.95)	**0.003**
	Public	12 (15%)	226 (95%)	—	—	—	—

City of residence	Amman	112 (17.8%)	517 (82.2%)	1.78 (1.20–2.63)	**0.004**	1.52 (1.01–2.29)	**0.043**
	Other cities	39 (10.9%)	320 (89.1%)	—	—	—	—

Number of languages spoken by the child	One language	86 (11.2%)	680	3.27 (2.27–4.72)	**0.000**	2.18 (1.47–3.22)	**0.000**
	Two or more languages	65 (29.3%)	157				

Siblings of the child	One or no siblings	72 (24%)	228 (76%)	2.43 (1.71–3.47)	**0.000**	1.69 (1.16–2.46)	**0.006**
	Two or more siblings	79 (11.5%)	609 (88.5%)	—	—	—	—

Mother’s education	Mid school or high school	21 (18.4%)	93 (81.6%)	1.29 (0.78–2.15)	0.32	—	—
	College diploma or a higher degree	130 (14.9%)	744 (85.1%)	—	—	—	—

Child’s daily frequency of tooth brushing	Frequent	112 (19.2%)	472	2.22 (1.51–3.28)	**0.000**	1.61 (1.07–2.43)	**0.024**
	Infrequent	39 (9.7%)	365				

Previous caries‐related dental visit	Yes	71 (13.2%)	465 (86.8%)	1.41 (1.00–1.99)	0.053	—	—
	No	80 (17.7%)	372 (82.3%)	—	—	—	—

Previous checkup‐related dental visit	Yes	55 (22.3%)	192 (77.7%)	1.93 (1.33–2.78)	**0.000**	1.22 (0.82–1.83)	0.33
	No	96 (13%)	645 (87%)	—	—	—	—

Child has a positive attitude to a dental appointment	Yes	39 (22.2%)	137 (77.8%)	1.78 (1.18–2.68)	**0.006**	1.21 (0.76–1.90)	0.42
	No	112 (13.8%)	700 (86.2%)	—	—	—	—

Child has a negative attitude to a dental appointment	Yes	32 (11.4%)	249 (88.6%)	1.58 (1.04–2.39)	**0.033**	1.34 (0.85–2.12)	0.20
	No	119 (16.8%)	588 (83.2%)	—	—	—	—

Belief in Tooth Fairy	Yes	96 (100%)	0 (0%)	2.46E + 10	—	—	—
	No	55 (6.17%)	837	—	1.00	—	—

*Note:* Statistically significant results are shown in bold (*p* < 0.05) to attract attention of readers.

The CFA was carried out to examine cross‐cultural validity. A five‐factor model provided a good fit to the data. The five‐factor model included the following: first: sociodemographic and child‐related variables, second: dental factors, including dental visits, third: factors related to the child’s experience of shedding the first primary tooth, fourth: emotions experienced by the child when the first tooth started to wiggle and when this tooth was lost (two items only), fifth: all questions about the Tooth Fairy. The fit indices were as follows: TLI = 0.990, CFI = 0.991, GFI = 0.995, and RMSEA = 0.051. However, it should be noted that this model was built based on one previous research and our understanding of the included factors. Future research may explore alternative factor structures or examine the stability of the factor model across different populations. The Cronbach’s alpha was 0.7.

## 5. Discussion

This study was conducted to investigate the different emotions experienced by children aged 5–8 years upon losing the first primary tooth, as reported by parents. To the best of our knowledge, this study represents the second study in the dental literature and the first in Jordan to shed light on emotions experienced by children upon the loss of the first primary tooth.

The minimum sample needed for the study was 721. However, to allow generalization of the results of the study, the sample size was increased. The final sample was 988 parents. An online questionnaire was completed by parents from different cities in Jordan using social media to reach the targeted sample. This study established an acceptable validity and reliability of the questionnaire used.

The results of the present study show clearly that when losing the first primary tooth by typical shedding, more children experience positive rather than negative emotions. A similar conclusion was reached by Patcas et al. [6]; however, in their study, they reported a higher percentage rate of positive (82%) and a lower percentage rate of negative emotions (22%). In a recent study conducted in Jordan to evaluate the impact of early childhood caries on the quality of life of 4‐ and 5‐year‐old preschool children, besides pain, which was the most reported sign, irritability or frustration was also reported [21]. The floor effect was high on self‐image and social interaction, and child psychology in the child impact section [21]. This could be linked to the less positive and more negative emotions observed in this study than in the previous study in relation to any dental event that reminds the child of a previous unpleasant dental history, including the shedding of teeth. Also, cultural differences may have contributed to the dissimilarity in reported emotions. Patcas et al. [6] conducted their study in Switzerland, highlighting the difference in the reported positive emotions between children of parents originating from different countries and religions. They found that children of parents from non‐Western countries showed significantly more positive emotions than children from Western countries. Although explaining the finding is complicated, they suggested that the difference could arise due to diverse cultural rituals accompanying the shedding of the first primary tooth [6]. As various cultural parental norms elicit distinct responses in children [22], Patcas et al. [6] stated that it is reasonable to assume variations in children’s emotions depending on the significance of the tooth shedding being approved by parental culture. In addition, there are cultural dissimilarities in parent–child interaction and parenting style, which are not related to particular cults and customs [23]. It is the parental and dentist’s obligation to recognize possible influences on the child’s emotional development. Yet parents and dentists should be cognizant that physiologic tooth loss is predominantly associated with negative emotions for some children, which are not easy to modify [6]. Efforts should be directed toward creating a positive feeling at the time when teeth are starting to show signs of shedding.

This study reported significant negative emotions in children attending public schools. Children of families of middle and low socioeconomic class usually attend public schools in Jordan. Previous studies have demonstrated the impact of social class on anxiety displayed by children; socioeconomic status disparities profoundly impact child development and health, which are linked to negative emotions [24, 25].

In this study, the number of languages the child speaks has a statistically significant association with positive emotions experienced. The more languages the child speaks, the more positive emotions are expected to be expressed by the child. It was shown that bilingual fluency has benefits related to higher self‐esteem [26] and emotional and behavioral benefits [27].

Positive emotions significantly increased when the child had a check‐up‐related dental visit and when the child demonstrated a positive attitude toward dental treatment. Likewise, negative emotions decreased when the child was happy to visit the dentist and increased when the child was unhappy during the dental visit. In our study, dental caries and dental trauma as causes of dental visits were not predictors of positive or negative emotions. Patcas et al. [6] explained that positive emotions are decreased due to previous caries‐related visits, possibly because these past tooth‐related experiences might be associated with blame (of insufficient care), shame, or feelings of guilt. Earlier accident‐related visits, however, represent a potentially beneficial factor and increase the likelihood of positive emotions. In contrast to the previous dental trauma, an unexpected, abrupt, and painful experience often accompanied by a sense of loss of control and uncertainty, the gradual process of the anticipated shedding must be perceived as less sudden and painful [6]. The increased positive emotions related to check‐up dental visits and positive attitude to a dental visit found in the present study are in line with what was found by Patcas et al. [6] in that it confirms the importance of previous tooth‐related experiences and past challenges and their influence on children’s emotions.

In their review, Bögels and Brechman‐Toussaint [28] found that later‐born children tend to be more anxious and shy and might be dominated in a negative way by their older siblings, negative domination may justify the higher anxiety in later‐born children. Also, in larger families, more chaotic and conflictual family life may arise, and parents might not correct negative sibling domination [28]. In this study, it was confirmed that less negative emotions were reported by children who had one or no siblings compared to children with two or more siblings, and more positive emotions were related to the child having one or no older siblings compared to two or more older siblings.

A pathway proposed by Rachman [29] relates to acquiring fears through social processes. This pathway posits that children will learn to be fearful due to the negative information they have seen or heard from parents, family members, peers, teachers, television, or social media. Dental fear and anxiety may, therefore, develop as a result of exposure to negative information about dentistry/dental procedures [30]. However, siblings’ experiences with their modeling role may play an important role, either positively or negatively, in shaping children’s perception of the same event [31]. It seems that if a child witnesses tooth exfoliation in other children, it will affect a child living the same experience. Our results confirm that children are more likely to experience positive emotions and less likely to experience negative emotions if they have witnessed tooth exfoliation in older siblings or friends. This approach is utilized in the behavior management of dental anxiety in pediatric patients [32]. Most behavior modification is considered a preventive strategy through modeling because it is too late to truly intervene after the anxious child has arrived for their dentist appointment. Children seeing their siblings reacting well to dental care has been shown to counter the expected disruptive behavior of a fearful child [32].

In the present study, while only the parental educational level was associated with the children’s either positive or negative emotions, maternal education had only an association with telling the child about the Tooth Fairy. A previous study found that maternal and paternal education was one of the determinants of positive emotions, but the negative emotions were only associated with the father’s education [6]. Against these findings, in the present study, the father’s education was not a predictor of experiencing positive or negative emotions upon losing the first primary tooth. It has been reported that time spent by fathers on their children is significantly affected by the father’s educational background; the greater quality of father–child interactions and relationships can anticipate positive socioemotional adaptation in children [33]. Many studies found that more fathers with a high school diploma spent time in reading activities with their toddlers than those without a high school diploma [34, 35]. Although fathers’ involvement in the education and development of their young children is a growing phenomenon, unfortunately, it is still lacking in shared reading, especially in the Middle East and North Africa region [36]. This might be connected to the cultural role of the father within Jordanian society, as the father is considered the main or only income earner in a household and has power, while the mother is considered the caregiver.

To help children cope with the experience of the natural loss of a primary tooth and to make it as positive as possible, it is important to acknowledge the validity of the child’s feelings. To reduce the intensity or effect of the negative feeling, parents may find a way to celebrate the occasion, and many families opt to have fun with the Tooth Fairy tale. Parents are more likely to tell their children about the Tooth Fairy when their children are attending private and international schools. In Jordan, private and international schools are usually attended by children of parents from a high socioeconomic background. The higher socioeconomic status among these families creates a higher chance of using the Tooth Fairy and exchanging the tooth for money. Children attending private and international schools and their parents are supposed theoretically to be exposed to cultures other than the local culture through multiple media (i.e., TV and internet). The highest prevalence of parents telling their children about the Tooth Fairy was in Amman, the capital of Jordan. According to the distribution of Jordan’s population, this can be seen as a logical result since about half of Jordan’s population lives in Amman. More facilities are present in the capital and, as it is, it includes most of the international schools, private schools, and universities. Also, it’s well known that the capital is more open to other cultures than other cities in the country.

More attention could be given to the child with fewer siblings, granting parents awareness of the significance of tooth loss and efforts to lessen its impact on their children. This study showed that the likelihood of telling the child about the Tooth Fairy was increased when the child had one or no siblings.

Against the findings reported by Patcas et al. [17], who found that teeth‐brushing frequency had no effect on the likelihood of a Tooth Fairy visit [17], in our study, brushing‐teeth frequency had a significant effect on the likelihood of a Tooth Fairy visit. The finding of Patcas et al. [17] was related to cultural background, whereas the finding in this study might be linked to parents’ education.

Almost every country in the world has a tradition related to the loss of primary teeth, including an act carried out by the child. These traditions are usually about the future, advocating a new tooth’s growth and strong development or focusing on the child’s overall health [12]. Such traditions help the child progress from early to later childhood [37, 38]. In addition, children who believe in magical figures, including the Tooth Fairy, comfort their parents that their child is still young and has not grown up so rapidly [1]. About one‐third of parents in our study told their children to throw their teeth into the sun. This tradition of throwing teeth to the sun or the sky was reported previously in Jordan, as well as in Northern Africa and other Middle Eastern countries, including Iraq, Palestine, Egypt, and Sudan [1, 39–41]. Parsons et al. [39] reported seven general themes for traditions practiced worldwide. These traditions have deep historical roots, demonstrating meaningful, physiological, and social functions for both the child and the parents [12]. In other words, these traditions have nonobvious aims, like addressing or even moderating certain unconscious psychological concerns that the children themselves are having trouble dealing with [1]. In this study, a low percentage of parents told their children about the Tooth Fairy, which is much lower than that reported by Patcas et al. [17], who found that most parents reported that the Tooth Fairy visited their children. The difference in the results might be ascribed to cultural differences since the Tooth Fairy is not a concept in the culture of the Jordanian population.

In light of the present study, parents and dentists are encouraged to increase their knowledge to approach tooth shedding in children, especially when noticing a tooth wiggle. When addressing a loose tooth with a pediatric patient, the healthcare professional can use culturally relevant language to describe how the tooth will fall out and how a permanent one will grow [12]. Knowing a patient’s background and needs helps to reduce anxiety and other negative emotions while enhancing trust in dental treatment [42]. Dental anxiety may be reduced when dentists are more familiar and can communicate harmoniously with patients according to their culture and family traditions, including the traditions of tooth loss [12].

This study had limitations related to online surveys. As was planned, we started to distribute the questionnaire to a random sample of schoolchildren, but unfortunately, due to the COVID‐19 pandemic and the lockdown during the time of the study, the survey was delivered online. Parents were reached from the authors’ social media groups. As parents who received the online form were asked to pass the electronic link of the questionnaire to other parents with the same inclusion criteria, the number of persons who received and read the questionnaire without completing it is unknown. Thus, the response rate could not be achieved. Additionally, emotions were not investigated using a specific scale measurement. Also, there was no control over the time that passed from the tooth shedding till the parents filled out the survey, and the precision of emotions recalled is not guaranteed. While we acknowledge the potential for recall bias inherent to the cross‐sectional design and the use of a questionnaire that possibly could have been subject to information bias. Although some time may have passed since losing the first primary tooth, the authors believe the significance of this event ensures greater memory retention among parents. Another possible limitation is the lack of studies between which results can be compared and differences explained.

## 6. Conclusion

This study demonstrates that the loss of the first primary tooth is a significant emotional milestone for children, eliciting a spectrum of both positive and negative responses. The nature of these emotions is significantly influenced by the child’s social and educational environment, including school type, multilingualism, and family structure—specifically the presence of older siblings and previous exposure to tooth exfoliation in others. Furthermore, prior dental visits were found to be a key determinant of a child’s emotional resilience during this transition. While the “Tooth Fairy” tradition is relatively uncommon in Jordan, its prevalence is linked to socioeconomic and behavioral factors such as residency and oral hygiene habits. To foster positive experiences, dentists and parents should proactively prepare children as soon as tooth mobility begins, ensuring that dental visits are utilized as opportunities to build emotional readiness for this natural developmental stage.

## Author Contributions

Lamis D. Rajab conceived the study and supervised the work. Lyn AE. El‐Smadi collected data. Both Lamis D. Rajab and Lyn AE. El‐Smadi analyzed the data, wrote and revised the manuscript.

## Funding

No funding was received to conduct this research.

## Disclosure

All authors have read and approved the final manuscript.

## Ethics Statement

Ethical approval was obtained, as previously described in the Materials and Methods section. IRB reference 75/2019/2284 date 31/11/2019.

## Conflicts of Interest

The authors declare no conflicts of interest.

## Data Availability

The data that support the findings of this study are available from the corresponding author upon reasonable request.
